# Trait-Based Dimensions Discriminating Adults with Attention Deficit Hyperactivity Disorder (ADHD), Autism Spectrum Disorder (ASD) and, Co-occurring ADHD/ASD

**DOI:** 10.3390/brainsci11010018

**Published:** 2020-12-26

**Authors:** Artemios Pehlivanidis, Katerina Papanikolaou, Kalliopi Korobili, Eva Kalantzi, Vasileios Mantas, Dimitra Pappa, Charalambos Papageorgiou

**Affiliations:** 1 1st Department of Psychiatry, Medical School, National and Kapodistrian University of Athens, “Eginition” Hospital, 72-74 Vas. Sofias Ave, 11528 Athens, Greece; lina.korompili@gmail.com (K.K.); kalantzieva@yahoo.gr (E.K.); mantas.vasilios@yahoo.com (V.M.); Pap1dimitra@gmail.com (D.P.); chpapag@med.uoa.gr (C.P.); 2 Department of Child Psychiatry, Medical School, National and Kapodistrian University of Athens, “Agia Sophia” Children’s Hospital, 11527 Athens, Greece; katpapan@med.uoa.gr

**Keywords:** attention deficit hyperactivity disorder (ADHD), autism spectrum disorder (ASD), adults, self-report dimensions

## Abstract

This study assessed the co-occurrence of attention deficit hyperactivity disorder (ADHD) and autism spectrum disorder (ASD) in newly diagnosed adults of normal intelligence and the contribution of trait-based dimensions deriving from the Barkley Adult ADHD Rating Scale-IV (BAARS-IV), the Autism-Spectrum Quotient (AQ), and the Empathy Quotient (EQ) to the differentiation of patients with ADHD, ASD, and ADHD/ASD. A total of 16.1% of patients with ADHD received a co-occurring ASD diagnosis, while 33.3% of patients with ASD received an ADHD diagnosis. Subjects with ADHD or ADHD/ASD had higher scores in all ADHD traits compared to ASD subjects. Compared to the ADHD group, the ASD group had AQ scores that were significantly greater, except for attention to detail. ADHD/ASD co-occurrence significantly increased the score of attention to detail. The total EQ score was greater in the ADHD group. In the stepwise logistic regression analyses, past hyperactivity, current inattention and impulsivity, attention switching, communication, imagination, and total EQ score discriminated ADHD patients from ASD patients. Attention to detail, imagination, and total EQ score discriminated ADHD cases from ADHD/ASD cases, while past hyperactivity and current impulsivity discriminated ASD subjects from ADHD/ASD subjects. Our findings highlight the importance of particular trait-based dimensions when discriminating adults with ADHD, ASD, and co-occurring ADHD/ASD.

## 1. Introduction

Attention deficit hyperactivity disorder (ADHD) and autism spectrum disorder (ASD) are amongst the most prevalent neurodevelopmental disorders. ADHD is characterized by developmentally inappropriate inattention, impulsiveness, and/or hyperactivity that remain relatively persistent over time and result in impairment across multiple domains of life activities. ASD is characterized by persistent deficits in social interaction and communication, as well as restrictive, repetitive patterns of behavior or interests [1]. These disorders typically have a childhood onset and persist into adult life, but there is a significant unmet clinical and research need to understand the persistence into adulthood [2,3,4,5,6,7]. In clinical practice, a notable symptom overlap between ASD and ADHD symptoms may lead to the diagnostic overshadowing of ADHD symptoms in ASD and vice versa. Despite the fact that prior to the fifth edition of the Diagnostic and Statistical Manual of Mental Disorders (DSM-5) [1], a diagnosis of ASD precluded a diagnosis of ADHD, there has been a considerable amount of research focusing on the co-occurrence between ADHD and ASD and many questions have been posed about the relationship between the two disorders. So far, the body of literature is, for the vast majority, based on childhood data. Research data show that 18 to 50% of children with ADHD present with clinical levels of ASD symptoms [7,8,9,10]. Conversely, ADHD is the most common co-occurring disorder in children with ASD, with co-occurrence rates in the 40–70% range [11,12,13]. Moreover, it has been reported that the overshadowing of symptoms in co-occurring cases that had been diagnosed with ADHD first may lead to a delay in the diagnosis of ASD [14]. Research data on the co-occurrence of ADHD and ASD in adulthood are scarce. The proportions of current or past diagnoses of ADHD in ASD adult patients have been reported to be from 9.7 to 43%, with most reports being within the range of 37–43% [6,15,16,17,18,19], while Edvinsson et al. [20] found that over 10% of a sample of patients diagnosed with ADHD in adulthood had a current or past diagnosis of ASD. Some researchers have even categorized ADHD and ASD as falling on the same continuum of neurodevelopmental disorders, with ADHD representing a less severe presentation of ASD [21,22].

Although carrying out a diagnosis following the DSM or International Classification Diseases (ICD) criteria has been the norm in child and adult psychiatry, a dimensional or hybrid model approach has been adopted for polythetic syndromes [23]. There is increasing recognition that dimensions of behavior can cut across diagnostic categories and those dimensions can be helpful in successfully categorizing individuals with neurodevelopmental disorders. Consistent with the hypothesis that co-occurrence of ADHD and ASD is better examined using dimensional traits are studies that investigate mechanisms underlying the co-occurrence of the two disorders. Polderman et al. [24] investigated specific patterns in the co-occurrence of ASD and ADHD traits in adults. Five trait-based dimensions of ASD (social skill impairments, strong routine preferences, attention-switching problems, imagination impairments, and a strong fascination for numbers and patterns), and two dimensions of ADHD (inattentive (IA) and hyperkinetic/impulsive (HI)) were jointly examined in a population-based adult sample. HI problems did not correlate substantially with the ASD trait dimensions, whereas IA problems correlated only with the ASD dimension assessing attention-switching difficulties. Furthermore, Polderman et al. [25] examined the genetic and environmental etiology of the association between specific ASD and ADHD disorder dimensions. In a community sample of adult twins, they assessed self-reported data on the ASD social and communication difficulties (ASDsc) and repetitive and restricted behavior and interests (ASDr) dimensions and the ADHD IA and HI dimensions. They concluded that ASDr problems form an important link between ASD and ADHD comorbidity. Ghirardi et al. [26] studied individuals aged 20–28 years from the Swedish Study of Young Adult Twins. They estimated the phenotypic and etiological overlap between self-rated trait dimensions of ADHD and ASD. At the phenotypic level, HI was correlated more strongly with repetitive and restricted behaviors (RRB) than social interaction and communication (SIC), whereas IA was equally associated with both dimensions of ASD traits. Their findings suggest a dimension-specific phenotypic and etiological overlap between ADHD and ASD traits.

It has also been reported that ADHD prevalence in ASD decreases with age and preliminary data suggest that the association between ASD and ADHD traits may be somewhat lower in adulthood than in childhood/adolescence [27]. Hartman et al. [28] reported that both ADHD and ASD symptom constellations are not at all stable across development, with some symptom dimensions (attention problems and social problems) being much more persistent than other symptom dimensions (hyperactivity/impulsivity and repetitive behaviors). In a meta-analysis of social cognition [29], it was reported that the developmental trajectories of social cognition probably differ between ADHD and ASD as social cognitive deficits in ADHD might improve with age in most individuals. Lai and Baron-Cohen [6] stated that ADHD is seen in up to 40% of adults with ASD and support the view that the important point of differentiation is the nature of surface-level inattentiveness. ASD inattentiveness is characterized by a slowness or difficulty in attention disengagement and switching, whereas in ADHD, the main characteristic is difficulty in maintaining focused attention. They suggest that a dimensional perspective can be adopted.

The concept of empathy includes the identification of another’s mental state and the appropriate response to their mental state [30]. Abnormalities in empathy have been reported in various psychiatric disorders [31,32,33]. Individuals with ASD have repeatedly been reported to show impairments in empathy across the life span relative to typical controls [30,34] and in their ability to maintain selective attention when it concerns emotional stimuli [35]. The notion of empathy has not been adequately studied in adult patients with ADHD. There has been only one study [36] reporting that adults with a subclinical ADHD diagnosis had reduced levels of the Empathy Quotient (EQ) scores compared to the control group. To our knowledge, there are no reports on the possible role of empathy in diagnosing co-occurring ADHD and ASD in adults.

Data from studies that try to estimate the phenotypic and etiological overlap between self-rated trait dimensions of ADHD and ASD derive from a general population sample and, as such, the results might not be extrapolated directly to clinical settings. Clinicians performing an ASD/ADHD assessment are able to use their judgment to distinguish between ASD/ADHD and other conditions that can mimic ASD/ADHD symptoms [6] but often struggle to decide which disorder better describes the patient’s problems. They also acknowledge that when ASD and ADHD co-occur, each becomes more difficult to diagnose than when they occur in isolation [37]. Antshel et al. [11] comment that the base rate of ADHD symptoms for children, adolescents, and adults with ASD has never been firmly established and the extent to which these symptoms are inherent to ASD is not clear. Establishing symptom profiles for making a diagnosis of ADHD in individuals with ASD remains an important area of research. Of particular interest is the less studied group of the less impaired adult patients who are overlooked until adulthood and pose complex diagnostic issues [38].

As part of a comprehensive assessment of adults, self-rated questionnaires are often used. They can be useful either for screening purposes or in support of the diagnostic procedure. We hypothesize that individual trait dimensions that derive from questionnaires with widely accepted psychometric properties may offer helpful information for the differential diagnosis of adults with normal intelligence that are referred with a suspected ADHD, ASD, or ADHD/ASD diagnosis.

The aim of the present study was twofold: first, to estimate the rate of co-occurrence of ADHD and ASD in adults with normal intelligence that were referred for the first time in their lives to an Adult Neurodevelopmental Department, and second, to examine how the total score and individual trait dimensions, such as the current and past inattention, hyperactivity, and impulsivity that are derived from the Barkley Adult ADHD Rating Scale-IV (BAARS), the five subscales deriving from the Autism-Spectrum Quotient (AQ), and the total score of the EQ could help a clinician in everyday clinical practice to identify variables that could differentiate patients with ADHD, ASD, and ADHD/ASD. We particularly focused on the extent to which these trait dimensions can be helpful in disentangling the most puzzling group of patients with a dual ADHD/ASD diagnosis.

## 2. Materials and Methods

### 2.1. Recruitment

The present study was part of a larger research project on de novo diagnosed adults with ADHD and ASD [38]. The research group included 238 adults that were assessed at the Adult Neurodevelopmental Department in the 1st Department of Psychiatry at the National and Kapodistrian University of Athens and received a de novo ADHD and/or ASD diagnosis during a three-year period. The Adult Neurodevelopmental Department is the only nationwide center that accepts referrals from the general population across Greece for the diagnosis of ASD and ADHD in adults. It used to be specialized for the diagnosis and treatment of adult ADHD [38,39], but during the last ten years, the increased needs for diagnosis and treatment of adults with ASD resulted in the broadening of referral criteria in order to include suspected ASD cases. The multidisciplinary team that carries out all assessments consists of psychiatrists who have extensive experience in the diagnosis and treatment of neurodevelopmental disorders in adults and are trained in Autism Diagnostic Observation Scale (ADOS) [40,41], Autism Diagnostic Interview –Revised (ADI-R) [42,43], and Diagnostic Interview for ADHD in Adults (DIVA) [3,5,44]; clinical psychologists; a speech and language therapist; an occupational therapist; a social worker. In order to be included in the study, subjects had to be adults with normal intelligence and fluent phrase speech that received an ADHD and/or ASD diagnosis for the first time in their life. Exclusion criteria were a previous ADHD and/or ASD diagnosis, the presence of acute psychopathology requiring urgent psychiatric treatment, IQ < 70 according to the Wechsler Adult Intelligence Scale (WAIS), and a known genetic cause.

### 2.2. Procedure

The assessment procedure was built on a standard diagnostic routine that is described in detail in Pehlivanidis et al. [38]. Initially, all referred patients had to complete a questionnaire that comprised demographic, educational, occupational, and clinical questions and a battery of screening instruments, including modified versions of the BAARS-IV [45], the AQ [46], and the EQ [47]. The completed questionnaire was sent via e-mail to our clinic.

Responses to the questionnaire were then discussed with the patient and a family member when possible. Patients underwent a thorough psychiatric examination by experienced psychiatrists in our department by exploring the existence and pervasiveness of a suspected neurodevelopmental disorder and the presence of co-occurring psychopathology. If needed, patients were referred to other members of the multidisciplinary team or for a laboratory workout. The semi-structured interview DIVA was administered to all patients, while the ADOS and ADI-R were administered to selected cases that were considered to be more complicated. The final diagnosis regarding the presence of ADHD and/or ASD was given during a consensus meeting of the multidisciplinary team and was based on the DSM-5 criteria while taking into consideration all available information.

### 2.3. Measures of ADHD and ASD Dimensions

#### 2.3.1. BAARS-IV

A modified version of the BAARS-IV was used in order to assess self-reported ADHD traits. This 18-item questionnaire assesses the DSM-IV criteria for ADHD, with clinical response and recovery cutoffs that are derived from population-based norms. The BAARS-IV shows adequate internal consistency, test–retest reliability, and strong construct validity and discriminant validity [45]. It comprises 18 items for both current (last 6 months) and childhood (for age 5–12) behavior, using a four-point (0, 1, 2, 3) rating scale. A score of two or above indicates the presence of a symptom. Nine items assess the presence of inattentive symptoms and nine assess the presence of hyperactive/impulsive symptoms. For the needs of our study, we divided the hyperactivity/impulsivity items into two subscales: impulsivity (four items) and hyperactivity (five items). The following ADHD traits subscales were entered into the analysis: inattention for age 5–12 (0 to 9 points), hyperactivity for ages 5–12 (0 to 5 points), impulsivity for ages 5–12 (0 to 4 points), inattention in the last 6 months (0 to 9 points), hyperactivity in the last 6 months (0 to 5 points), impulsivity in the last 6 months (0 to 4 points), total score for ages 5–12 (0 to 18 points), and total score for the last 6 months (0 to 18 points). In our sample, Cronbach’s α for all scales was between 0.70 and 0.91.

#### 2.3.2. Autism Quotient

The AQ was originally developed to identify ASD among adults with normal intelligence [48]. It consists of 50 items, 10 items per subscale. The participants responded to each of 50 statements on a four-point Likert scale (1—“strongly agree,” 2—“slightly agree,” 3—“slightly disagree,” 4—“strongly disagree”). The total AQ, as well as the five subscale scores, namely, social skills (SS), attention switching (AS), attention to detail (AD), communication (C), and imagination (I), were calculated using the relevant key. Woodbury-Smith et al. [49] were the first to evaluate the AQ for its potential as a screening questionnaire in clinical practice and concluded that the AQ is strongly predictive of who receives a diagnosis of ASD in normal intelligence and high-functioning adults. Other researchers [50] questioned the utility of the AQ for predicting ASD caseness in patients seen at an adult ASD diagnostic service when using the total AQ score. Moreover, in a more recent analysis [51], it was suggested that several measurement properties of the AQ are good and have adequate sensitivity and specificity to distinguish people with ASD from those without ASD but noted that the AQ cannot be described as a unidimensional measurement. In our study, the following five AQ subscales were entered into the analysis: social skills, attention switching, attention to details, communication, imagination, and the total AQ score. In our sample, Cronbach’s α for the total score and the subscales were between 0.73 and 0.84, except for the imagination and attention switching subscales, where Cronbach’s α was 0.59 and 0.50, respectively.

#### 2.3.3. Empathy Quotient (EQ)

The EQ is a self-assessment instrument for measuring the construct of empathy in adults of normal intelligence, both as a one-factor and a three-factor dimension [52,53,54]. It comprises 40 questions that examine empathy and 20 filter items [35,47]. Responses are given on a four-point Likert scale and scores can range from 0 to 80 points. Reduced levels of EQ scores reflecting deficits in empathy have been repeatedly reported for individuals with ASD, relative to typical controls [55]. The EQ is inversely correlated with the AQ [55]. The EQ has been validated in a Greek adult population, demonstrating good psychometric properties and supporting both the one- and the three-factor models [56]. In the present study, the total EQ score was entered into the analysis. Cronbach’s α was 0.71.

### 2.4. Statistical Analysis

The normal distribution of the variables was evaluated using the Kolmogorov–Smironov test. Qualitative variables are expressed as absolute and relative frequencies. The chi-square test was used for the comparison of proportions. For the comparison of continuous variables between the three study groups, one-way analysis of variance (ANOVA) was used. A Bonferroni correction was applied in the case of multiple testing in order to eliminate type I errors. Multiple logistic regression analyses with a stepwise method (*p* for removal was set at 0.1 and *p* for entry was set at 0.05) were also performed in order to identify the variables that could differentiate the studied disorders. Adjusted odds ratios with 95% confidence intervals were computed from the results of the logistic regression analyses. We performed a series of multiple logistic regression analyses with dependent variables that were used to define the groups with different disorders. All reported *p*-values are two-tailed. Statistical significance was set at *p* ≤ 0.05 and the analyses were conducted using SPSS statistical software (IBM SPSS Statistics Base 22.0).

## 3. Results

The sample consisted of 238 participants (172 men and 66 women) with a mean age of 30.2 years (SD = 9.8 years). A total of 151 subjects (63.4%) were diagnosed with ADHD, 58 (24.4%) with ASD, and 29 (12.2%) with both ADHD and ASD. Of the total number of patients that received an ADHD diagnosis, either as a sole or co-occurring diagnosis (*n* = 180), 29 (16.1%) received a comorbid ASD diagnosis, while of the 87 subjects diagnosed with ASD, 29 (33.3%) fulfilled the criteria for an ADHD diagnosis. Sample characteristics of all groups are shown in Table 1. The three groups of patients were similar in terms of sex and age (Table 1).

The self-reported measures of ADHD subscales for the three study groups are shown in Table 2. As expected, subjects with ADHD had significantly higher scores on all ADHD traits compared to the ASD subjects. Furthermore, subjects having both ADHD and ASD diagnoses had significantly greater scores on all ADHD traits compared to ASD ones. No significant differences concerning ADHD trait scores were found between subjects with ADHD and those having both ADHD and ASD.

A comparison of the self-reported measures of AQ and EQ scores between the three study groups are presented in Table 3. Scores in the AQ traits were found to be significantly greater in the ASD subjects compared to the ADHD subjects, as expected, with the exception of attention to detail. Additionally, all AQ trait scores were found to be significantly greater in those having both ADHD and ASD in comparison to those having ADHD alone. Furthermore, the attention to detail score was found to be greater in those having both ADHD and ASD compared to those with ASD. The total EQ score was greater in ADHD subjects compared to ASD cases and those that had both ADHD and ASD.

The results from the stepwise logistic regression analyses (Table 4) showed that hyperactivity at ages 5–12, inattention in the last 6 months, impulsivity in the last 6 months, attention switching, communication, imagination, and the total EQ score could discriminate ADHD patients from ASD patients. Furthermore, multiple analyses showed that attention to detail, imagination, and the total EQ score could discriminate ADHD cases from those having both ADHD and ASD, while hyperactivity at ages 5–12 and impulsivity in the last 6 months were found to significantly discriminate between ASD and ADHD/ASD subjects.

## 4. Discussion

In our study, we estimated the rate of co-occurrence of ADHD and ASD in patients referred to an Adult Neurodevelopmental Department and explored the dimensions of self-rated screening instruments that could help a clinician discriminate between patients with ADHD, ASD, and ADHD/ASD.

### 4.1. Co-occurrence of ADHD and ASD

Among the 238 normal intelligence adults that were assessed during a three-year period, 63.4% received an ADHD diagnosis only, 24.4% received an ASD diagnosis only, and 12.2% received a dual ADHD/ASD diagnosis. A total of 16.1% of the 180 patients with an ADHD diagnosis received a co-occurring ASD diagnosis, while of the 87 subjects diagnosed with ASD, 33.3% fulfilled the criteria for an ADHD diagnosis. Our findings are in line with previous findings showing elevated ratings of ADHD symptoms in ASD patients and elevated but lower rates of ASD symptoms in ADHD patients. Anckarsäter et al. [57] reported that from 260 consecutive adults that were assessed for current and lifetime ADHD and ASD, 113 subjects had ASD and 147 had ADHD, while in 46 (19.2%), there was an overlap between ASD and ADHD. In a naturalistic study in an adult psychiatric population, from the 84 patients diagnosed with ASD, 37% fulfilled the criteria for comorbid ADHD [19]. In Hofvander et al.’s study [15], an ADHD clinical diagnosis was given to 43% of 122 adult outpatients with ASD. Johnston et al. [16] reported that from the 31 adults with ASD and average intellectual function who completed self-report measures of ADHD symptoms, 36.7% met the DSM-IV criteria for current ADHD “caseness.” Lai and Baron-Cohen [6] stated that ADHD is seen in up to 40% of adults with ASD. The only study showing low rates of comorbid ADHD in ASD adult patients is a retrospective case review [18], where only 9.7% of those who met the ICD-10 criteria for ASD received a diagnosis of ADHD, a rate that was not significantly greater compared to the general population data. However, the authors commented that there was an associated specialist ADHD clinic within the same set of services and many referrals were possibly diverted away from the ASD clinic. Regarding co-occurring ASD in adults with ADHD, a study [20] examining the prevalence and gender differences of DSM-IV axes I and II comorbidity in a clinical cohort of patients diagnosed with ADHD in adulthood found that over 10% of the sample had a current or past diagnosis of ASD, which is comparable to the rate of 16.1% found in our study.

### 4.2. BAARS Dimensions

Patients with a confirmed ADHD diagnosis scored higher in the total number of self-reported ADHD symptoms compared to ASD patients, as expected, but not higher compared to ADHD/ASD patients. The same applied to all ADHD trait subscales (Table 2). In the stepwise logistic regression analysis (Table 4), the ADHD traits subscale scores that could discriminate ADHD patients from ASD were hyperactivity at ages 5–12, inattention in the last 6 months, and impulsivity in the last 6 months. Furthermore, the scores of hyperactivity at ages 5–12, together with impulsivity in the last 6 months, could discriminate ADHD/ASD patients from ASD patients. It seems that the following four ADHD items that describe current impulsivity—(1) talked excessively, (2) blurted out answers before questions had been completed (completed others’ sentences or jumped the gun), (3) had difficulty waiting their turn, and (4) interrupted or intruded on others (butted into conversations or activities without permission or took over what others were doing)—may be very useful for the discrimination between adults with a dual ADHD/ASD diagnosis and those patients having ASD as a sole diagnosis. The fact that impulsivity at ages 5–12 was not among the discriminant factors could be explained by the suggestion made by Barkley [45] stating that symptoms of impulsivity, especially verbal impulsivity, begin to emerge as a semi-distinct dimension of ADHD symptoms in adulthood and is not so distinct from hyperactivity in children’s ratings or in the retrospectively recalled symptoms of childhood, as reported by adults. Furthermore, in accordance with our findings, Barkley et al. [58] noted that in a free interview context, patients’ responses for the symptoms (1) making decisions impulsively and (2) having difficulty stopping activities or behavior when they should do so are the best discriminating symptoms for ADHD from other forms of psychopathology. The above symptoms were also found useful in order to screen for ADHD among anxious and depressive adult psychiatric outpatients [39]. There are very few disorders apart from ADHD where impulsivity is part of the diagnostic criteria, such as borderline personality disorder or some obsessive-compulsive spectrum disorders [1,59]. Therefore, current impulsivity may be viewed as a red flag for a possible co-occurring ADHD in patients with other diagnoses. Impulsivity is also considered to play a central role in understanding ADHD/ASD comorbidity. Sokolova et al. [60] studied the relationship between ASD and ADHD symptoms by applying causal modeling. They used a large phenotypic dataset of 417 children with ASD and/or ADHD, 562 affected and unaffected siblings, and 414 controls to infer a structural equation model using a causal discovery algorithm. The strongest links they found were between social communication difficulties, inattention, and impulsivity, and suggested that impulsivity has a causative effect on social ineptness.

In clinical practice, the finding that self-reports of current impulsivity can discriminate ASD patients from ASD/ADHD patients has both diagnostic and treatment implications. Interviewing adults of normal intelligence with an ASD diagnosis for current impulsivity may lead to a co-occurring ADHD diagnosis, which has to be treated accordingly (i.e., with stimulants).

### 4.3. AQ Dimensions

As expected, the total AQ score and most of the AQ subscale scores were higher in adults with ASD compared to adults with ADHD, with the exception of the subscale score of attention to detail. Furthermore, patients with ADHD/ASD scored higher in all AQ scores compared to patients with ADHD.

When comparing patients with ASD to patients with ADHD/ASD, no difference was found, with the exception of the subscale attention to detail, whose scores were higher in the combined group. It seems that attention to detail scores did not significantly differ between ADHD and ASD, but the co-occurrence of the two disorders significantly increased the score in this subscale. Furthermore, stepwise logistic regression multiple analysis showed that attention to detail, along with imagination, were two of the three dimensions (the third one being EQ score) that discriminated ADHD/ASD cases from those having ADHD, with imagination being lower, while attention to detail being higher in the dual-diagnosed group.

Our results for “attention to detail” are in line with previous reports separating this subscale from the other subscales of AQ. In confirmatory factor analyses in both a general population and a student sample [61], four out of the five domains of the AQ (social skill, communication, attention switching, and imagination) were highly correlated. The authors proposed a hierarchical model, allowing these four domains to cluster together as a ‘‘social interaction’’ factor, while a small second factor, consisting of items focusing on a preference for details and patterns (the domain ‘‘attention to detail’’) was also identified. The usefulness of the AQ in differentiating between adult ASD and adult ADHD was also studied by Sizoo et al. [62], who explored whether substance use disorder (SUD) comorbidity affects AQ scores. Once more, the total AQ score and most of the AQ subscale scores were higher in adults with ASD compared to adults with ADHD, except for the subscale score attention to detail. The attention to detail subscale is composed of items referring to a perceptual style with a preference for details and patterns. It might be that this autistic dimension scale does not discriminate between patients with ASD and patients with ADHD because it refers to a strategy for dealing with aspects of attention deficit that is common to both disorders. Patients with ASD present attention deficit distress when they are overwhelmed by perceptual stimuli. The distress is said to decrease when focusing on logical sequences, such as patterns, telephone numbers, or car license plates. On the other hand, patients with ADHD might try to compensate for their attention deficits by focusing on patterns and details. It seems that the co-occurrence of ADHD/ASD further increases the attention deficit and consequently a stereotyped behavior that is registered as an increased score in the attention to detail subscale. Attention to detail could also be an underlying psychological mechanism that explains why ASD repetitive and restricted behavior and interests seem to form a link between ASD and ADHD [24,26].

Imagination was the other dimension deriving from the AQ that differentiated individuals with ADHD from individuals with ASD or ADHD/ASD, indicating that a lack of imagination could be considered a discriminating trait in adults with ASD, regardless of ASD being a sole or co-occurring diagnosis. It is well known that people with ASD form a group of individuals for whom spontaneous and fantastical acts of imagination appear to be a challenge. Children with autism do not engage in spontaneous pretend play in the ways that typically developing children do, engaging instead in repetitive activities, and adults with autism are less interested in fiction [63,64,65,66].

### 4.4. EQ Score

Τhe EQ total score was lower in ASD and ADHD/ASD patients compared to ADHD patients, while no difference was found between ASD and ADHD/ASD patients (Table 3). Furthermore, stepwise logistic multiple regression analysis showed that the EQ score was among the factors that discriminated both ASD and ADHD/ASD cases from ADHD cases (Table 4). It seems that a low EQ score, similarly to low imagination, is indicative of the presence of ASD either as a sole diagnosis or as a co-occurring disorder.

The EQ has not been systematically studied in adults with clinical ADHD diagnosis. Groen et al. [36] reported reduced EQ scores in adults with a subclinical DSM-5 ADHD diagnosis compared to the control group, although still within the normal range. They considered that the reduced EQ score may be related either to a reduced emotion regulation/emotional lability in patients with ADHD or ASD comorbidities. The latter is supported by our finding that EQ was reduced only in the group with ADHD/ASD co-occurrence and not the ADHD group.

The EQ is designed to measure how easily a person can detect other people’s feelings and is affected by them. Since empathy is a core skill that facilitates effective social interaction [30], a lower empathetic ability may reflect less social adaptability and may be the key factor in understanding reasons for referrals in adulthood [67]. Lower empathizing traits, as measured by the EQ in individuals that are referred and diagnosed in adulthood, may be especially important in understanding challenges with social adaptability. Measures assessing social cognition may not be sensitive enough to detect difficulties in functioning for adults of normal intelligence because of the “camouflaging” of ASD-related characteristics in social situations where a patient is motivated by the desire to fit in with others [68].

The finding that EQ discriminates adults of normal intelligence with co-occurring ADHD/ASD from patients with ADHD has important clinical implications. Interviewing for the capacity to detect other people’s feelings when assessing adults for a possible ADHD diagnosis might reveal symptoms indicating the possible co-occurrence of ASD. Treating ADHD with co-occurring ASD is much more complex than treating ADHD as a sole diagnosis.

### 4.5. Limitations

A number of limitations of this study should be taken into consideration. First, our subjects represent a specific clinical population, and thus, the results cannot be generalized to other samples, such as community or low-functioning samples. Second, the study population was not very large, especially the combined ADHD/ASD population. Third, we used structured interviews only in selected cases that were considered to be more complicated. However, previous research has shown that there is a moderate agreement between clinical diagnoses and ADOS4, while ADI-R might not be reliable in adults without an intellectual disability [69,70,71]. An extended psychiatric interview made by an experienced psychiatrist, combined with collateral information, is probably the most essential part of the diagnostic assessment in high-functioning adults. Furthermore, the BAARS-IV is based on the DSM-IV criteria for ADHD. Nevertheless, the 18 items of the BAARS-IV are similar in terms of the number and quality of the DSM-5 items and the BAARS-IV is the only screening instrument where the three dimensions of ADHD (inattention, hyperactivity, and impulsivity) are considered separately [45]. Another limitation of the study is the low Cronbach’s α for the attention switching and imagination subscales of the AQ. It is noteworthy that a low degree of internal consistency for some subscales of the AQ, and in particular for the imagination subscale, has been reported in previous studies too [61,72,73,74,75]. Therefore, findings regarding these particular dimensions should be considered with caution. Finally, researchers must always be prudent with the interpretation of results of self-reported questionnaires when used for individuals with neurodevelopmental disorders where self-reflection and metacognitive skills might be impaired. This is particularly an issue with people having an ASD examination, where a poor awareness of autism-related traits may lead to an under-reporting of autism symptoms and over-reporting of social competency [50,76].

## 5. Conclusions and Research Implications

Despite the limitations, our results illustrate that apart from considering diagnostic categories, individual trait-based dimensions deriving from screening instruments (BAARS-IV, AQ, and EQ) might be of particular help in the comprehensive assessment and differential diagnosis of adults of normal intelligence with a suspected ADHD or ASD diagnosis, and in particular, of those with the more perplexing ADHD/ASD diagnosis. Being able to successfully categorize individuals with ADHD, ASD, or ADHD/ASD by using clinical dimensions is an important first step toward identifying the atypical brain function and structure underlying these clinical features. Our findings highlight the importance of the dimensions of current impulsivity, attention to detail, imagination, and empathy when discriminating adults with ADHD, ASD, and co-occurring ADHD/ASD. They also suggest the need to study the neural underpinnings of these particular traits using a lifespan approach in order to understand the persistence and co-occurrence of ADHD and ASD in adulthood.

## Figures and Tables

**Table 1:** Sample characteristics by group.

	Diagnosis			Test Statistic (df) *	*p*
Demogrsphics	ADHD *N* = 151	ASD *N* = 58	ADHD + ASD *N* = 29		
Age (years), mean (SD)	31.0 (10.0)	28.7 (9.2)	28.8 (10)	1.41 (2, 234)	0.246
Sex, *N* (%)					
Men	106 (70.2)	47 (81)	19 (65.5)	3.21 (2)	0.201
Women	45 (29.8)	11 (19)	10 (34.5)		

* F (df_1_, df_2_) for ANOVA, *χ*^2^ (df) for Pearson’s chi-square test. ADHD: attention deficit hyperactivity disorder, ASD: autism spectrum disorder.

**Table 2:** Comparison of the self-reported number of ADHD traits between the three study groups.

	ADHD	ASD	ADHD + ASD	F (df_1_, df_2_) *	*p* ^#^			
	A	B	C			A vs. B	A vs. C	B vs. C
ADHD Traits	Mean (SD)	Mean (SD)	Mean (SD)			*p* ^#^	*p* ^#^	*p* ^#^
Inattention at ages 5–12 years	6.2 (2.4)	3.9 (2.6)	6.6 (2.5)	20.00 (2, 229)	<0.001	<0.001	>0.999	<0.001
Hyperkinetic at ages 5–12 years	2.7 (1.6)	1.5 (1.6)	3.1 (1.6)	15.36 (2, 229)	<0.001	<0.001	0.716	<0.001
Impulsivity at ages 5–12 years	2.1 (1.4)	1.1 (1.3)	2.6 (1.3)	15.35 (2, 229)	<0.001	<0.001	0.360	<0.001
Total score at ages 5–12 years	11 (4.4)	6.4 (4.4)	12.4 (4.3)	27.90 (2, 229)	<0.001	<0.001	0.399	<0.001
Inattention in the last 6 months	5.8 (2.5)	3.1 (2.6)	5.5 (2.9)	22.75 (2, 231)	<0.001	<0.001	>0.999	<0.001
Hyperkinetic in the last 6 months	2.7 (1.5)	1.6 (1.4)	2.9 (1.5)	12.56 (2, 231)	<0.001	<0.001	>0.999	0.001
Impulsivity in the last 6 months	1.8 (1.4)	0.8 (1)	2.2 (1.5)	15.12 (2, 231)	<0.001	<0.001	0.328	<0.001
Total score for the last 6 months	10.3 (4.3)	5.4 (4)	10.6 (5)	27.17 (2, 231)	<0.001	<0.001	>0.999	<0.001

* F (df_1_, df_2_) for ANOVA; ^#^ *p*-values after multiple comparisons using a Bonferroni correction.

**Table 3:** Comparison of the self-report number of ASD traits between the three study groups.

	Diagnosis			F (df_1_, df_2_) *	*p* ^#^			
	ADHD	ASD	ADHD + ASD					
	A	B	C			A vs. B	A vs. C	B vs. C
ASD Traits	Mean (SD)	Mean (SD)	Mean (SD)			*p* ^#^	*p* ^#^	*p* ^#^
Social skills score	4 (2.4)	6.1 (2.5)	6 (2.4)	19.25 (2, 223)	<0.001	<0.001	<0.001	>0.999
Attention switching score	5.8 (1.8)	6.8 (1.7)	6.9 (1.8)	9.38 (2, 223)	<0.001	0.001	0.010	>0.999
Attention to detail score	4.8 (2.4)	4.7 (2.4)	6.3 (2.1)	7.84 (2, 223)	<0.001	>0.999	0.008	0.020
Communication score	4.5 (2.4)	5.8 (2.6)	6.7 (2.1)	13.19 (2, 223)	<0.001	0.001	<0.001	0.412
Imagination score	3.6 (1.9)	5.3 (2.2)	5.1 (2.1)	17.94 (2, 223)	<0.001	<0.001	0.001	>0.999
AQ total score	22.7 (6.2)	28.9 (6.9)	30.9 (7.2)	30.22 (2, 223)	<0.001	<0.001	<0.001	0.529
Empathy total score	31.7 (10.5)	23.4 (10.1)	19.7 (6)	26.61 (2, 223)	<0.001	<0.001	<0.001	0.307

* F (df_1_, df_2_) for ANOVA; ^#^ *p*-values after multiple comparisons using a Bonferroni correction.

**Table 4:** Results from the stepwise logistic regression analysis for the discrimination of ADHD, ASD, and ADHD/ASD groups of patients from the self-reported number of ADHD and ASD traits.

Self-Reported Traits Discriminating Groups	OR (95% CI) *	*p*
***ADHD vs. ASD***		
Hyperkinetic at age 5–12	1.68 (1.22–2.32)	0.001
Inattention in the last 6 months	1.50 (1.22–1.84)	<0.001
Impulsivity in the last 6 months	1.77 (1.1–2.84)	0.019
Attention switching score	0.68 (0.51–0.9)	0.008
Communication score	0.76 (0.6–0.97)	0.025
Imagination score	0.74 (0.59–0.92)	0.007
Empathy quotient	1.08 (1.02–1.15)	0.012
***ADHD + ASD vs. ADHD***		
Attention to detail score	1.25 (1.00–1.55)	0.050
Imagination score	1.38 (1.09–1.74)	0.007
Empathy quotient	0.87 (0.81–0.93)	<0.001
***ADHD + ASD vs. ASD***		
Hyperkinetic at age 5–12	1.72 (1.22–2.42)	0.002
Impulsivity in the last 6 months	2.13 (1.41–3.24)	<0.001

* Odds ratio (95% confidence interval).

## Data Availability

Data available on request due to privacy restrictions.
